# Internalised Weight Stigma Moderates the Impact of a Stigmatising Prime on Eating in the Absence of Hunger in Higher- but Not Lower-Weight Individuals

**DOI:** 10.3389/fpsyg.2019.01022

**Published:** 2019-05-08

**Authors:** Angela Meadows, Suzanne Higgs

**Affiliations:** ^1^School of Psychology, University of Exeter, Exeter, United Kingdom; ^2^School of Psychology, University of Birmingham, Birmingham, United Kingdom

**Keywords:** weight stigma, self-stigma, internalised weight stigma, eating behaviour, eating in the absence of hunger

## Abstract

A considerable body of evidence links internalised weight stigma with higher levels of disordered eating behaviour and cognitions in both normative- and higher-weight populations. However, to date, the impact of internalised weight stigma on objectively measured food intake has not been explored. In the present study, a weight-diverse sample of 158 non-smoking adults (BMI ≥ 25 kg/m^2^ *n* = 72, BMI < 25 kg/m^2^ *n* = 86) were recruited to a study on “The effects of hunger and satiety on information processing.” Participants first completed a series of online questionnaires, then attended a lab visit in a fed state. Participants were randomised to read a sham news article on the negative consequences of either weight (stigma condition) or smoking (control condition) and answer some questions about the article. Then, under the pretence of a non-study-relevant break, participants were exposed to a pre-weighed selection of sweet and savoury snacks for 15 min. Mood and hunger levels were assessed prior to and after reading the vignette, and after the break. In contrast to the relationship with self-report eating behaviour, internalised weight stigma was not a significant independent predictor of total energy intake and did not moderate the relationship between exposure to the stigma prime and calories consumed. However, differences emerged on the basis of participants’ weight status. Higher-weight participants with high levels of internalised weight stigma consumed fewer snack calories following exposure to a weight-stigma prime compared with a neutral prime (*B* = −137, *SE* = 58, *t* = −2.35, *p* = 0.020, 95% CI −252, −22) whereas those with low levels of internalised weight stigma tended to eat more in the weight stigma condition (*B* = 118, *SE* = 62, *t* = 1.91, *p* = 0.059, 95% CI −4, 241). In normative-weight participants, no differences in energy intake by levels of internalised weight stigma were observed. These findings suggest differences in the relationships between internalised weight stigma and self-reported disordered eating behaviour versus eating in the absence of hunger (EAH) measured under laboratory conditions. Additionally, internalised weight stigma appears to have differential effects on response to stigma in higher-weight and normative-weight individuals.

## Introduction

Higher-weight individuals face prejudice and discrimination in employment, education, healthcare settings, and a wide range of everyday interpersonal situations (Puhl and King, 2013). In addition to being the target of weight stigma from others, some individuals internalise society’s anti-fat attitudes and stereotypes – that is, they devalue themselves because of their weight (Durso and Latner, 2008; Lillis et al., 2010). Studies have consistently found associations between both experiences of weight stigma and internalised weight stigma and a wide range of problematic eating behaviour in both adults and children, even after controlling for body mass index (BMI), self-esteem, mood disorders, and other potential confounds (for reviews, see Menzel et al., 2010; Nolan and Eshleman, 2016; Vartanian and Porter, 2016; Pearl and Puhl, 2018). Internalised weight stigma also appears to mediate the relationship between experiencing stigma from others and downstream problematic eating (Durso et al., 2012; O’Brien et al., 2016; O’Hara et al., 2016). However, the majority of the literature linking weight stigma with eating behaviour consists of cross-sectional studies using entirely self-report measures. While there are obvious pragmatic reasons for this, attempts should be made to confirm findings using measures of objective eating behaviour, and to utilise experimental designs that allow for determination of causal mechanisms.

To date, only four studies have explored the impact of exposure to weight-related stigmatising material on actual energy intake (Schvey et al., 2011; Major et al., 2014; Mulder et al., 2015; Shentow-Bewsh et al., 2016), with conflicting results. In a lab-based study of 34 “overweight” and 39 “normal-weight” females, fasted subjects watched either a weight-stigmatising or a neutral video, before being given access to a large amount of snack food (Schvey et al., 2011). The “overweight” women in the stigma condition ate over three times as many calories as those who watched the neutral video, and significantly more than “normal-weight” women in either video condition. In another study, Major et al. (2014) randomised 93 fasted female university students to read a sham news article about how either weight or smoking status could negatively impact on employment prospects, which was partly explained by greater healthcare insurance costs for higher-weight or smoking employees, who were deemed more likely to suffer ill health. On subsequent exposure to snack foods, self-perceived “overweight,” but not “non-overweight” women in the stigma condition consumed more calories than those in the neutral condition. The authors proposed that this effect was driven by social identity threat, which occurs when an individual is reminded or made aware that a group they belong to is socially devalued (Steele et al., 2002; Major and O’Brien, 2005). Coping with such threats to one’s social identity can involve a range of strategies, including suppression of negative emotions or attempts to present oneself more positively (Miller and Kaiser, 2001), all of which require effortful self-control, and which have been demonstrated to deplete the cognitive resources required for subsequent self-control, for example, when presented with highly palatable but “unhealthy” snack foods (Hofmann et al., 2009). However, the vignettes used in the study by Major et al. (2014) discussed both employment and health problems often associated with being higher-weight; it is therefore not possible to determine whether subsequent eating behaviour was being driven by weight-related social identity threat or by non-identity related stress arising from more pragmatic concerns around actual or potential health or employment problems.

Also using a self-relevant threat paradigm, Mulder et al. (2015) exposed undergraduate students to one of three sham magazine articles about “obesity,” which included either a moralising discourse about “obesity,” a counter-moralising discourse about “obesity,” or a control condition with no moralising or counter-moralising content. The dependent variable was choice of a healthy versus unhealthy snack post-experiment. Broadly speaking, across two experiments, counter-moralising arguments tended to induce greater healthy snack choice in higher-weight individuals, but more frequent unhealthy snack choice in lower-weight individuals. Statistical analyses were not performed on the control versus moralising condition, but data on percentages choosing healthy snacks suggest that higher-weight individuals exhibited similar or slight increase in healthy snack choice in the moralising condition compared with the control condition. Findings for lower-weight individuals suggested either increase, decrease, or no difference between the two conditions and are thus difficult to interpret (Mulder et al., 2015). It should be noted that given the pervasiveness of anti-“obesity” messages in society, even the supposedly neutral article – which noted the rising prevalence of “obesity” – may have implied moralisation and so unintentionally induced threat, which could explain the generally minor differences in snack choice between the control and moralising conditions among high-weight individuals. Thus, these studies may not provide a true comparison between exposure to a stigmatising versus a non-stigmatising stimulus.

A more recently published study randomised 120 weight-diverse female undergraduates to either a weight-stigma condition or one of two control conditions (Shentow-Bewsh et al., 2016). In the weight-stigma condition, participants read a sham newspaper article about the “obesity epidemic” that portrayed the burden to individuals and the economy of higher-weight peoples’ poor choices, repeated several negative stereotypes about higher-weight individuals, and gave first-person accounts of interpersonal stigma experiences. In a subsequent taste-rating task of high-caloric snacks, lower-weight participants tended to eat more in the weight-stigma condition than in the control conditions, although the effects were small and not statistically significant. In contrast, higher-weight participants tended to eat more than lower-weight participants in both of the control groups, but did not differ in energy intake from their lower-BMI counterparts after reading the “anti-obesity” article, suggesting that exposure to this stigmatising prime was causing them to moderate their food intake. One possible explanation is that higher-weight participants were engaging in impression-management behaviour – that is, eating in such a way as to produce a more positive impression on others (Vartanian et al., 2007; Vartanian, 2015). The salience of the stigmatised identity in the weight-stigma prime condition may have prompted heavier individuals, whether consciously or unconsciously, to engage in stereotype-relevant self-presentation techniques (Neel et al., 2013), in this case, moderating their snack intake in order to counter stereotypes that higher-weight individuals are greedy and lacking in self-control (Allon, 1982; Puhl et al., 2005; Lewis et al., 2010).

Importantly, none of these studies explored the role of participants’ own internalised weight stigma in determining their response to weight-based stigma or identity threat. As noted above, a considerable body of evidence now links internalised weight stigma with patterns of disordered eating behaviour and cognitions in diverse populations, both independently, and as a mediator of the relationship between experienced weight stigma and maladaptive eating. Thus, an understanding of the impact of internalised weight stigma on objective eating behaviour may be of importance in developing effective individual and public health interventions aimed at tempering non-physiological energy intake. It is possible that exposure to societal weight stigma may have differential effects depending upon the degree to which an individual has previously internalised weight stigma. Thus, the deleterious effect of weight stigma may be particularly pronounced in a person who believes that stigma is deserved and appropriate, whereas an individual with low internalised weight stigma may discount a stigmatising experience as simply an indicator of prejudice in the perpetrator, with no detrimental impact on their own self-worth, and a consequently reduced or even null effect of the stigma on eating behaviour compared with high internalisers.

Thus, the present study sought to explore the impact of a weight-related stigma prime on food intake under laboratory conditions and the potential moderating role of internalised weight stigma in a weight-diverse sample of adult men and women. As noted above, the studies by Major, Schvey, and colleagues both used fasted subjects. The findings from these studies likely represent the phenomenon of eating more than needed to satisfy hunger when exposed to weight-related stigmatising situations, and may have more ecological validity for predicting excessive intake at meal times. The study by Shentow-Bewsh et al. (2016) did not use fasted or fed subjects, but participants were more hungry than full. However, people frequently eat when they are not hungry. An alternative measure of non-physiological energy intake is the Eating in the Absence of Hunger (EAH) paradigm, which is perhaps more comparable with the concept of hedonic eating. EAH studies are usually conducted in two stages: participants are first allowed to eat until sated, before being told that a short break is required prior to the second part of the study. During this break, participants are given access to either a second meal or a large amount of highly palatable snack foods, with energy intake at this point being the outcome of interest (Fisher and Birch, 2002). In a number of stress-manipulation studies conducted using the EAH paradigm, participants consumed an *ad libitum* meal and were then randomised to complete either a simple or an unsolvable maths puzzle, intended to increase stress and anxiety, prior to the break period (Rutters et al., 2009; Lemmens et al., 2011). These studies found that stress increased subsequent EAH in both “normal weight” and “overweight” adults, particularly those with higher levels of disinhibited eating; however, the effect was significantly amplified in “overweight” participants (Lemmens et al., 2011).

In the present study, we first explored the impact of internalised weight stigma on energy intake following exposure to a weight-related stigmatising prime or a neutral prime. We utilised an interpersonal relationship paradigm, whereby the stigmatising prime discussed the detrimental impact of high-weight status on personal relationships. This paradigm was intended to situate the stressor specifically within a social identity setting, without incurring potential non-identity related stress associated with economic or health concerns in general. We predicted that individuals higher in internalisation would eat more following exposure to the stigma prime than those low in internalisation. We further explored whether this relationship would be moderated by participants’ objective or self-classified weight status – that is, would the relationship differ for individuals with higher-weight versus normative BMI and/or self-classified “overweight” versus “non-overweight”^1^. Three contrasting but plausible outcomes could be predicted for the three-way relationship between experimental condition, internalised weight stigma, and weight status on energy intake. First, higher-weight individuals with higher levels of internalised weight stigma could experience more distress in the weight stigma condition and engage in non-physiological eating behaviour as a coping mechanism – i.e., eating more in response to stigma exposure compared with those lower in internalised weight stigma, consistent with the findings from self-report measures, and more than lower weight participants, consistent with findings from laboratory studies of objective eating behaviour. Alternatively, higher-weight individuals with elevated internalised weight stigma would be both more aware and more ashamed of their stigmatised status, and engage in impressions management behaviour, consuming fewer calories. If this were the case, we would expect a significant three-way interaction between condition, internalised weight stigma, and weight status such that objective or self-classified “overweight” participants with high levels of internalised weight stigma would eat less in the stigma condition compared with the control condition. Finally, it was possible that levels of internalised weight stigma would capture most of the variance associated with being higher-weight and exposed to stigma, in which case, we would expect no significant three-way interaction between condition, internalised weight stigma, and weight status. Thus, this second analysis was considered exploratory, and no *a priori* hypothesis was proposed.

## Materials and Methods

### Sample

Community and student participants were recruited for a study on “the effects of hunger and satiety on information processing” using a mix of social media, an online classified advertisement website, a free United Kingdom portal for the recruitment of research participants, the university website and departmental noticeboards, a database held by the School of Psychology of individuals who had previously expressed an interest in participating in research, and from the School’s Research Participation Scheme. Eligibility requirements were age 18–69 years, a never-smoker, no food allergies, and no eating disorder diagnosis. Additionally, to ensure recruitment across the BMI spectrum, some advertisements were targeted to recruit higher-weight participants, with the additional eligibility requirement that individuals self-classify as being “overweight.” The social media channels included groups related to dieting, fitness, healthy living, plus-size fashion, body image, size acceptance, and general interest groups linked to the local area. The use of these different sites was intended to attract a diverse range of higher-weight participants whose feelings about their size might vary between being more positive or negative. Participants recruited through the School of Psychology Research Participation Scheme received course credit for taking part in the study. Other participants were entered into a prize draw to win a £30 gift voucher and paid £5 for their time. The study was approved by the University of Birmingham Ethical Review Committee, and informed consent was obtained from all participants.

### Procedure

**FIGURE 1:**
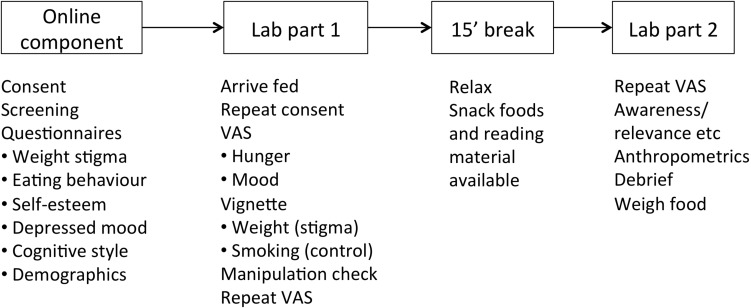
Schematic overview of study design.

The study was conducted in two stages, with the first stage completed online, and the second stage taking place in the laboratory (Figure 1). All computer-based portions of the study were conducted using the Qualtrics survey platform^2^. For the online component, after providing explicit consent, participants completed an initial screening and package of questionnaires, described below. The screening confirmed that participants were never-smokers, had no food allergies, and had not been diagnosed with an eating disorder. Any participants who did not pass the screening were thanked for their time and exited from the study. On completion of the online portion of the study, participants were emailed and informed that they had been randomised to attend the lab session “full,” and were provided a link to an online poll with timeslots available in the morning and afternoon. They were asked to choose a slot as close as possible to the time they usually finished eating either breakfast or lunch.

On arrival at the laboratory, consent forms and allergy cheques were repeated. Participants were asked to confirm that they had recently eaten a meal; those who had not (*n* = 16) were not excluded, but this information was noted and explored as a possible covariate. Participants were then led to a private room with a computer monitor, and a separate table, chair, some magazines, and a selection of pre-weighed sweet and savoury snack foods. Magazines were selected that did not contain any content or advertising relating to food, weight, or health. Participants were informed, “I’ll return in about 20 min. We’ve got some magazines in case you finish early. Help yourself to snacks – there’s plenty.” Participants were then left alone to complete the questions on the computer. First they were prompted to enter their unique ID code and reminded that this maintained the anonymity of their responses. Participants were then presented with five visual analogue scales (VAS) related to hunger (Hungry, Full, Thirsty, Desire to eat, Amount they could eat) and five related to mood (Anxious, Relaxed, Happy, Drowsy, Alert). Scales were anchored at 0 and 100 and participants dragged a marker along the scale to indicate their current state. They were then randomised to read either a weight stigma or control vignette, both written in the style of a newspaper article, describing the potential detrimental effects of either “obesity” or “smoking” on romantic relationships. This approach was taken to focus the threat at the level of interpersonal relationships, removing potential confounding effects of structural or institutional stigma. The experimental vignette described findings from scientific studies suggesting that being “obese” had a negative impact on perceived desirability as a dating partner. The description of the studies was taken from a review of research on weight stigma and interpersonal relationships (Puhl and Heuer, 2009). The article was completed by a fabricated “quote” from a fictional person belonging to a genuine United Kingdom-based relationship counselling charity. The quote related how “obesity” created interpersonal problems within existing relationships, even when the matter was not overtly discussed, thus ensuring the vignette was pertinent to individuals regardless of their current relationship status. The control vignette was identical with the exception that all words pertaining to weight were replaced with words pertaining to smoking. The vignettes used are available in the Supplementary Materials.

After reading the vignette, participants indicated whether they found the article easy to understand, interesting, and relevant to themselves. They were asked to briefly summarise the article, and then to provide any additional details they remembered. These questions served to support the cover storey, to ensure the details of the vignette were processed and recalled, and also acted as an attention check. Participants then repeated the hunger and mood VAS scales, and were shown a completion screen asking them to await the return of the researcher. Using the Qualtrics platform, the exact time of completion of each survey could be tracked. All participants were left for 15 min after completing the survey^3^, after which, the researcher returned and informed the participants that there were a few more questions to complete at the computer. Participants repeated the hunger and mood ratings, and finally, were probed for suspicion as to the true purpose of the study, if and when they had realised that we were interested in their snack consumption, and whether they thought the newspaper article had influenced what they ate. Finally, participants were debriefed, and anthropometric data collected. Height was measured using a stadiometer. Weight and percentage body fat were measured using a Tanita T5896 (Tanita Corporation, Tokyo). Measured height and weight were used to calculate BMI.

### Measures

Sample characteristics were determined using the following measures, which were completed online, prior to attending the lab visit. No forced responses were stipulated.

#### Internalised Weight Stigma

Internalised weight stigma was assessed using the modified version of the 11-item Weight Bias Internalisation Scale (WBIS-M; Pearl and Puhl, 2014), which assesses the extent to which participants devalue themselves because of their weight. While the original WBIS used the wording “because I am overweight,” the modified version replaces this with “because of my weight,” thus facilitating the use of the scale across the weight range. A sample item is: “Because of my weight, I don’t understand how anyone attractive would want to date me.” Items are scored from 1 (strongly disagree) to 7 (strongly agree), with a mean score calculated for the full scale. Higher scores indicate greater internalised weight stigma. The WBIS-M had strong internal reliability in a weight-diverse sample, and was strongly correlated with body dissatisfaction, and moderately correlated with disordered eating and psychopathology, controlling for BMI (Pearl and Puhl, 2014). It has been used in US and Australian samples (Pearl and Puhl, 2014; O’Brien et al., 2016). Psychometric properties of the WBIS-M are similar in individuals classified as “overweight” and “non-overweight” by both BMI and self-classification criteria (Lee and Dedrick, 2016). Cronbach’s α in the present sample was 0.94.

#### Experienced Weight Stigma

Prior experienced weight stigma was initially assessed using the Stigmatising Situations Inventory (SSI; Myers and Rosen, 1999), a 50-item questionnaire that measures experiences of weight stigma across 11 domains. However, initial reports of online survey access indicated high rates of attrition, with few participants completing the online portion of the study. In order to reduce participant burden, a decision was made to replace the 50-item SSI with a three-item measure that has been used in a number of studies in recent years (e.g., Puhl et al., 2011; Pearl et al., 2012; Pearl and Puhl, 2016; Himmelstein et al., 2017). Specifically, these questions ask whether participants have ever been teased, treated unfairly, or discriminated against because of their weight. Each question receives a yes or no response, giving a possible range of 0–3. For the sake of brevity, and to distinguish this measure of experienced weight stigma from the SSI, the name EWS-3 will be used in the present study. The EWS-3 has not been psychometrically validated, but has been shown to correlate positively with internalised weight stigma (Himmelstein et al., 2017) and support for anti-weight discrimination policies (Puhl et al., 2011; Puhl et al., 2017). Kuder-Richardson’s α in the present study was 0.67 (see section Experienced Weight Stigma Measures for further discussion).

#### Eating Behaviour

Two measures were used to assess eating habits. The Dutch Eating Behaviour Questionnaire (DEBQ; van Strien et al., 1986) comprises three subscales that look at habitual eating patterns: dietary restraint, emotional eating, and external eating – eating in response to external cues rather than bodily hunger signals. Items are scored on a 5-point Likert scale measuring frequency of the different styles of eating behaviours, ranging from 0 (never) to 5 (very often). The individual subscales are scored separately. Higher scores indicate more frequent disordered eating. The subscales of the DEBQ have good to excellent internal reliability in “obese” and “non-obese” men and women (van Strien et al., 1986), and has been validated in United Kingdom non-clinical samples of men and women and dieting and eating disordered women (Wardle, 1987). Although the factor structures are gender-invariant, women tend to score higher on the restraint and emotional subscales (Wardle, 1987). Cronbach’s α for the DEBQ Restraint, External Eating, and Emotional Eating subscales were 0.93, 0.86, and 0.94, respectively.

The Eating Disorder Diagnostic Scale (EDDS; Stice et al., 2000) was used to assess cognitions and behaviours consistent with eating pathology. Items are summed to produce a composite symptom count that can be used as a measure of overall eating pathology, with higher scores indicating more problematic cognitions and behaviours (Stice et al., 2004). The EDDS has good internal consistency in both clinical and non-clinical female samples, high test-retest reliability, excellent concordance with interview diagnoses of disordered eating, and good convergent validity with self-report measures of disordered eating behaviour and general psychopathology (Stice et al., 2000, 2004). While not formally validated in adult males, the EDDS also had strong internal reliability in a sample of male U.S. veterans, and scores were uniquely predicted by military trauma, controlling for other potential confounds (Arditte Hall et al., 2017). Questions relating to height and weight were omitted from the original 22-item scale, as this information was collected elsewhere. Thus, the final questionnaire included 20 questions. Cronbach’s α in the present sample was 0.81.

Additionally, current dieting behaviour was assessed with a single item. Participants indicated whether they were currently dieting for weight loss, watching their food intake so as to maintain their current weight and prevent weight gain, or not dieting.

#### Depressive Symptoms

As depressed mood may influence food intake, depressive symptomatology was assessed with the Center for Epidemiological Studies Depression Scale (CES-D; Radloff, 1977). This is a 20-item measure that assesses the frequency of affective and behavioural symptoms of depression over the previous week. Items are scored on a 4-point rating scale from 0 (rarely or none of the time) to 3 (most or all of the time), and a sum score is calculated for the whole scale. Higher scores indicate more depressive symptoms. The CES-D has high internal consistency, adequate test-retest reliability, and similar reliability, validity, and factor structure across demographic categories. Although not designed for clinical diagnosis, it has good discriminant validity between clinical and non-clinical populations, and correlates moderately with severity ratings in clinical patients (Radloff, 1977). Cronbach’s α in the present sample was 0.91.

#### Self-Esteem

Global self-esteem was measured with the Rosenberg Self-Esteem (RSE) scale (Rosenberg, 1965). The RSE is the most widely used measure of global self-esteem and has good internal and test-retest reliability and convergent, discriminant, and predictive validity (Donnellan et al., 2015). The RSE correlates negatively with measures of experienced and internalised weight stigma and disordered eating cognitions and behaviours (Griffiths et al., 1999; Friedman et al., 2005; Durso and Latner, 2008). Items are scored on a 4-point Likert scale ranging from 0 (strongly disagree) to 3 (strongly agree). The maximum possible score is 30, and higher scores are indicative of higher self-esteem. Cronbach’s α in the present sample was 0.89.

#### Need for Cognition

Finally, to support the cover storey – that the purpose of the study was to examine the relationship between hunger and satiety and information processing – and help disguise the actual focus of the study, subjects completed the 18-item Need for Cognition Scale (NCS), which assesses an individual’s tendency to engage in and enjoy effortful cognitive endeavours (Cacioppo et al., 1984). Items are scored from −4 (very strong disagreement) to +4 (very strong agreement), with higher scores indicating greater need for cognition. The scale has good reliability and convergent validity (Cacioppo et al., 1984; Tolentino et al., 1990). Cronbach’s α in the present sample was 0.88.

#### Anthropometrics and Demographics

Participants self-classified their weight on a 5-point scale: “Underweight,” “Normal weight,” “A little overweight,” “Moderately overweight,” or “Very overweight.” Self-classified weight status was dichotomised into self-classified “overweight” (those who indicated they were a little, moderately, or very “overweight”) and self-classified “not overweight” (those who indicated they were “underweight” or “normal weight”). Demographic data comprising age, gender, ethnicity, education level, and profession were collected.

### Eating in the Absence of Hunger Paradigm

Prior to each participant’s arrival at the laboratory, six identical small bowls were heaped full of a selection of three savoury and three sweet snack foods^4^. In total, the six bowls of snack foods provided approximately 4500 kilocalories and 200 g of fat. The bowls were weighed before and after the experimental session to determine the amount eaten. The number of grams of each type of snack food was converted into kilocalories, and summed to provide total energy intake.

### Data Analysis

All analyses were conducted using the SPSS for Mac Statistical Software package, version 24.0, unless stated otherwise.

#### Power Analysis

Prior to the start of the study, a power analysis was conducted with G*Power 3.1 (Faul et al., 2007). Given the difficulty in detecting moderator effects with continuous variables (Shieh, 2009), sample size was determined on the basis of the hypothesised three-way interaction between experimental condition, weight status, and internalised weight stigma. All simple effects, two-way, and three-way interactions, and the intercept were included in the analysis, and baseline hunger was included as a covariate. A sample of 146 participants would yield 80% power to detect a small-to-medium effect size (*f*^2^ = 0.085) for the tested predictors at the α = 0.05 significance level.

#### Handling of Missing Data

Missing data analysis was conducted on questionnaire responses. Four participants each had one data point missing, one participant skipped the entire RSE questionnaire and one skipped the DEBQ-Restraint and External subscales. Additionally, 14 participants (8.5%) did not have data for body fat percentage. All of these cases were due to practical considerations; no participants declined to be weighed and measured. Analysis of all study variables against outcome measures indicated that values were missing completely at random, Little’s MCAR test χ^2^(63) = 66.2, *p* = 0.37, therefore subsequent analyses were conducted with missing values excluded pairwise.

#### Preliminary Analyses

First, separate linear regression analyses were used to confirm that recruitment group (community versus student participants) was not a significant predictor of experienced or internalised weight stigma, or of total energy intake, after controlling for age, gender, and BMI. All analyses were non-significant; thus, groups were combined in subsequent analyses.

The proposed factor structure for the VAS was confirmed using principal components analysis with varimax rotation. Examination of the scree plot indicated two distinct factors, accounting for 54.6% of the variance. All hunger and mood VAS items loaded > 0.5 onto their respective factors. Items with negative loading were inverted and a mean mood and hunger score was calculated for each time point.

To confirm successful randomisation to weight stigma or control experimental condition, independent *t*-tests and $\chi^{2}$ test were used to compare distribution of demographic variables, scores on online questionnaire measures, and relevant baseline measures taken in the laboratory. Independent *t*-tests, univariate ANOVAs, and univariate linear regressions were used to explore whether potential confounds were significant predictors of energy intake. Repeated-measures ANOVAs were used to test change in hunger levels, overall mood, happiness, and anxiety by experimental condition and objective and self-classified weight status. Bonferroni correction was used to account for violation of the assumption of sphericity.

#### Main Analyses

The proposed interaction effect between experimental condition and participants’ internalised weight stigma on energy intake, was tested using PROCESS version 3.0 for SPSS, model 1 (Hayes, 2017). The potential three-way interaction between experimental condition, internalised weight stigma, and weight status was tested using PROCESS model 3. The PROCESS macro utilises a robust, non-parametric bootstrap resampling procedure with replacement to produce an unstandardised regression coefficient, and a bias-corrected 95% confidence interval (CI) for each predictor, with 5,000 bootstrap samples utilised in the present analyses. The HC3 estimator was used to ensure heteroscedasticity-consistent standard errors (Hayes and Cai, 2007). All continuous variables were mean-centred. Experimental condition was dummy coded as 1 = Weight-stigma condition, 0 = Control condition. Two measures of participants’ weight status were used: objective BMI category (coded ≥ 25 kg/m^2^ = 1, < 25 kg/m^2^ = 0) and self-classified “overweight” status (coded “overweight” = 1, “non-overweight” = 0). Interactions were interpreted by examining simple effects (Aiken and West, 1991). It is recommended that interaction effects be probed at meaningful values of the moderators (Hayes, 2017). Thus, for the dichotomous variables (experimental condition, BMI category, self-classified “overweight”), effects were tested at the two values of the moderator. For internalised weight stigma, slopes were tested at values of 2.5 and 5.5 (−0.9 and 2.1 after mean-centring), representing the lower and upper quartiles of the range of the scale.

## Results

### Sample Descriptives

Three hundred and twenty participants consented to take part in the study. Nineteen were screened out prior to beginning the survey (10 with food allergies, two smokers, and seven with a diagnosed eating disorder), and a further 12 exited the survey during the screening procedure. Of the 289 participants who began the online survey, 220 (76%) completed all questions and were invited to arrange a laboratory visit. Of these, 164 (75%) attended the lab-based phase of the study. Six participants failed the participation cheque during the lab-based component of the study – that is, they were unable to describe the contents of the vignette, indicating either lack of attention or lack of comprehension, and their data were excluded from further analyses, giving a final sample size of 158.

The sample was predominantly female (78.5%), and White (75.9%; Indian Asian/Asian British 8.9%, Black 3.8%, Chinese 3.2%, South-East Asian 1.9%, other ethnicity 2.5%, missing 3.8%), with a mean age of 26.0 years (*SD* 11.4, range 18–69 years). Three-quarters of the sample were students (75.9%),^5^ and 29.1% had an undergraduate or advanced degree. The BMI range for the sample was 14.8–58.2 kg/m^2^ (*M* = 23.3, *SD* = 6.1). Eight-six participants (54.4%) had a BMI < 25 kg/m^2^ and 72 (45.6%) had a BMI ≥ 25 kg/m^2^, however, 53.7% of participants self-classified as “overweight.”

### Experienced Weight Stigma Measures

Of the 158 participants included in the final sample, only eight had completed the 50-item SSI measure of experienced weight stigma. The remainder completed the EWS-3. Depending on the measure used, notable differences were observed in the proportion of participants who reported prior experience of weight stigma. Using the three-item EWS-3, only 38.7% participants endorsed any item. In contrast, using the SSI, all but one (87.5%) endorsed previous weight stigma experiences.^6^ Further, correlations between other study variables and EWS-3 were much lower than with SSI scores. Despite being used frequently, the EWS-3 appears to underestimate previous stigma experience, and findings using the two measures are unlikely to be comparable. As a result, and given that only eight participants in the final sample had completed the SSI, results for these two measures were not combined, and only the 150 participants completing the EWS-3 were included in subsequent analyses.

### Preliminary Analyses

Demographic variables did not differ by experimental condition. No differences were observed between experimental conditions in BMI, objective or self-classified “overweight,” dieting status, self-esteem, internalised weight stigma, depressive mood, need for cognition, self-reported eating behaviour, or baseline hunger and mood. Low baseline hunger levels confirmed the fed state. The percentage of participants who had previously experienced weight stigma was lower in the weight-stigma condition, with approximately one-third having prior stigma experiences, and two-thirds reporting no previous weight stigma experience. In the control condition, the breakdown was 50-50. Thus, experienced weight stigma was included as a covariate in subsequent analyses.

Energy intake did not differ by age, ethnicity, education, profession, time of experimental session, failure to eat prior to the session, dieting status, depressive symptoms, baseline mood, or reported ease of understanding, level of interest, relevance of the vignette, or awareness of true study intent. Statistical tests of energy intake by gender were non-significant, however, mean intake was noticeably different: male *M* = 201 kcals, *SD* = 225, female *M* = 136 kcals, *SD* = 151, *t*(41.5) = 1.60, *p* = 0.12, and lack of statistical significance may have been due to the much smaller sample size of male participants. Within gender groups, there was no difference in food consumption by experimental condition among male participants (*M* = 213 and 210 kcals in the control and weight-stigma conditions, respectively), however, mean intake in female participants was 158 kcals in the control condition and 91 kcals in the weight-stigma condition, *t*(57) = 1.7, *p* = 0.10^7^. Although this difference was not statistically significant, a conservative approach was taken and gender was included as a covariate in subsequent regression analyses.

Participants did not differ by experimental condition in how interesting or understandable they found the vignettes (both *p* > 0.6), however, more higher-weight participants in the weight-stigma condition reported that the vignette was personally relevant to them than did those in the control condition: 66.7% versus 33.3%, respectively, $\chi^{2}$(1) = 12.9, one-sided *p* < 0.00, Cramér’s *V* = 0.42. No differences in vignette relevance were observed for normative-weight participants.

Baseline hunger was a significant predictor of energy intake and was included as a statistical control in subsequent regression analyses. No changes in hunger were observed before and after reading the vignettes, but hunger decreased significantly after the food-available break period. Changes did not differ by vignette, weight status, or their interaction. No significant differences in overall mood, happiness, or anxiety were observed at any time point, and there were no differences by experimental condition, weight status, or their interaction. Repeating the analyses separately for those who ate or did not eat during the food-available period did not alter these findings. Overall, 27% of participants did not eat any of the snack foods, but this did not differ by experimental condition, weight status, or their interaction. As the distribution of dependent variables was negatively skewed due to the number of participants who did not eat any of the snack foods, the presence of extreme values was assessed visually using boxplots. A single outlier (weight-stigma condition) was identified: a male participant consumed 1,003 total kcal, with the range of remaining values falling between zero and 658 kcal. A conservative approach was taken whereby this value was replaced with the next highest intake by a male participant in the weight-stigma condition (653 kcal) to bring it closer to the distribution. Replacement of this extreme value in the moderation analyses resulted in small changes in model fit and regression coefficients, but did not alter the pattern of results.

### Main Analyses

**FIGURE 2:**
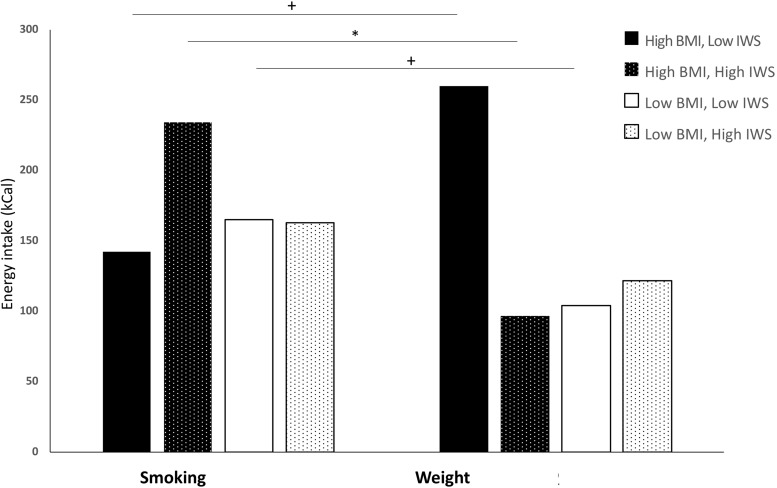
Impact of experimental condition on total energy intake by level of internalised weight stigma and weight status. High BMI ≥ 25 kg/m^2^; Low BMI < 25 kg/m^2^. High and low internalised weight stigma designated by upper (5.5) and lower (2.5) quartiles of modified Weight Bias Internalizstion Scale score range, respectively. BMI, Body mass index; IWS, internalised weight stigma. *p < 0.05. ${}^{+}$*p* < 0.08.

Baseline hunger, gender, and experienced weight stigma were entered as covariates in all models^8^. Contrary to expectations, moderation analysis with experimental condition, internalised weight stigma and their interaction as predictors of energy intake indicated no significant simple or conditional effects: experimental condition *B* = −41, *SE* = 25, *t* = −1.62, *p* = 0.108, 95% CI −91, 9; internalised weight stigma *B* = 15, *SE* = 13, *t* = 1.14*, p* = 0.255, 95% = −11, 42; interaction term *B* = −17, *SE* = 18, *t* = −0.92, *p* = 0.361, 95% CI = −53, 19. The full model, containing experimental condition, weight status by BMI category, internalised weight stigma, all two-way interactions, the three-way interactions, and all covariates, explained 28.4% of the variance in energy intake. Regression results are displayed in Table 1. The three-way interaction between experimental condition, internalised weight stigma, and BMI category was statistically significant, and explained 2.6% of the variance in energy intake. The conditional effect of internalised weight stigma on the relationship between experimental condition and energy intake was statistically significant in the high-BMI group only: *B* = −85, *F*(1, 139) = 7.46, *p* = 0.007. Simple effects analysis indicated that high-BMI participants with high levels of internalised weight stigma ate fewer calories in the weight stigma condition than in the smoking condition (conditional effect = −137, *SE* = 58, *t* = −2.35, *p* = 0.020, 95% CI −252, −22) whereas those with low levels of internalised weight stigma tended to eat more in the weight stigma condition (conditional effect = 118, *SE* = 62, *t* = 1.91, *p* = 0.059, 95% CI −4, 241 (Figure 2); contrast between conditional effect of experimental condition at high versus low internalised weight stigma *t* = 2.71, *p* = 0.008). The pattern of results was similar when weight status was defined by self-classified “overweight”, however, with the exception of baseline hunger, no significant simple or interaction effects were observed^9^.

**TABLE 1:** Effects of experimental condition, internalised weight stigma, and weight status on eating in the absence of hunger.

	*B*	*SE*	*t*	*p*	LLCI	ULCI
Constant	219	41	5.35	<0.000	138	299
Vignette	−55	36	-1.53	0.128	−126	16
IWS	−1	20	-0.03	0.976	−39	38
Weight status	5	41	0.12	0.902	−76	86
Vignette * IWS	7	27	0.25	0.801	−46	60
Vignette * Weight status	97	56	1.72	0.088	−14	208
IWS * Weight Status	31	28	1.12	0.266	−24	87
Vignette * IWS * Weight status	−92	41	-2.23	0.028	−173	−10
Gender	−70	36	-1.94	0.055	−141	1
Hunger	4	1	5.82	0.000	3	6
EWS	−18	17	-1.01	0.314	−52	17

**Unstandardised regression coefficients shown. Vignette coded 0 = Smoking, 1 = Weight; Weight status coded 0 = BMI < 25 kg/m^2^, 1 = BMI ≥ 25 kg/m^2^.EWS, Experienced weight stigma; IWS, Internalised weight stigma.**

## Discussion

This is the first study to explore the impact of internalised weight stigma on objectively measured eating behaviour. In contrast to consistently documented positive associations between internalised weight stigma and self-reported disordered eating behaviour in both normative- and higher-weight individuals (for a review, see Pearl and Puhl, 2018), no association was found between experimental condition, internalised weight stigma and EAH. Self-report measures capture habitual eating patterns over the longer term, and it is possible that in a given food-available situation, more immediate contextual influences may supersede any potential moderating impact by the factors that shape such behavioural tendencies. However, a significant three-way interaction between experimental condition, internalised weight stigma, and BMI status was observed. Among higher-weight, but not lower-weight participants, there were opposing trends based on levels of internalised weight stigma. Specifically, higher-weight participants with high levels of internalised weight stigma ate less in the weight-stigma condition than in the neutral condition. In contrast, unexpectedly, those low in internalised weight stigma tended to eat more when exposed to the weight-stigma prime, although this effect did not reach statistical significance.

A number of possible explanations may account for these results. In terms of reduced intake among higher-weight individuals with high levels of internalised weight stigma, one possibility would be that participants were motivated to represent themselves in a more positive light to mitigate others’ potential negative judgments. That is, individuals who feel high levels of guilt, shame, and self-devaluation based on their weight status may be more likely to engage in impressions management behaviour to counter stereotypes of greed and lack of willpower. An alternative possible mechanism underlying the unexpected reduction in energy intake in the weight-stigma condition among high-weight participants who were high in internalised weight stigma could involve conflicting goal motivational processes. Internalised weight stigma has been positively associated with dietary restraint and eating, weight, and shape concerns in some higher-weight community (Schvey et al., 2013) and treatment-seeking individuals (Almenara et al., 2017), although some studies have failed to find any such relationship (Lillis et al., 2010). Restrained eaters, i.e., chronic dieters, appear to be more responsive to environmental food cues, and under normal circumstances, hedonic drives may eclipse longer-term behavioural goals (for a review, see Papies et al., 2008). Under these circumstances, the availability of highly palatable, energy-dense snack foods would present a goal-conflict scenario, where the potential hedonic reward to be obtained from eating the food is incompatible with the desired weight-loss goal. However, priming goal-relevant information may inhibit conflicting goals and instigate conscious self-regulatory processes (Aarts and Dijksterhuis, 2000; Custers and Aarts, 2007). Thus, exposure to the weight-stigma vignette, which highlighted potential detrimental effects of “obesity” on interpersonal relationships, may have served to increase the salience of participants’ own weight-loss goals and behavioural intentions – goals more likely to be held by those high in internalised weight stigma (Puhl et al., 2018). Thus, in the present study, participants with high-BMI and high-internalised weight stigma may represent a group of high-restrained eaters. This hypothesis would also explain the relative rise in intake in this population assigned to the control condition; as weight was not made salient and thus weight-loss goals were not primed, intake would have been driven predominantly by the elevated hedonic reward associated with highly palatable foods.

While it is theoretically feasible that high levels of internalised weight stigma are acting as a proxy for high dietary restraint, which could explain why the high-BMI high-internalised weight stigma group specifically ate less in the weight stigma condition and more in the control condition, *post hoc* correlation analyses suggested that internalised weight stigma was associated with dietary restraint and current dieting behaviour in low-BMI participants only; the relationships were non-significant in high-BMI participants (see Supplementary Materials). Nevertheless, dietary restraint in the present study was measured with the Dutch Eating Behaviour Questionnaire (DEBQ), which is thought to identify more successful restrained eaters (van Strien, 1999; Stice et al., 2010). In contrast, the Restraint Scale (Herman and Mack, 1975; Herman and Polivy, 1980) is thought to capture unsuccessful restrained eaters (Heatherton et al., 1988; Williamson et al., 2007), and it is possible that use of an alternative measure of dietary restraint would have confirmed a strong relationship between internalised weight stigma and restraint in the high-BMI group.

A counterpoint to this hypothesis arises from an ecological momentary assessment study conducted in a community sample of 46 higher-weight men and women. Participants used personal digital assistant devices to record responses to perceived stigmatising experiences in real time over a 2-weeks period. Stigmatising incidents were associated with significantly lower momentary (and daily) motivation to diet, to exercise, and to lose weight in individuals with higher levels of internalised weight stigma compared with those who had lower internalised weight stigma (Vartanian et al., 2018). Nevertheless, there is evidence to suggest that where goal-conflict occurs, the presence of others is more likely to result in resisting the unwanted desire and decrease the likelihood that the goal-conflicting behaviour will be enacted – that is, to increase self-control (Hofmann et al., 2012). Thus, it is possible that, in the present study, the laboratory setting and the knowledge that any eating behaviour would be observable by the experimenter may have fortified self-regulatory behaviour in the presence of highly palatable snack foods.

It should also be noted that despite eating less during the study, high-weight participants with high levels of internalised weight stigma in the stigma condition may have engaged in a reactive episode of eating after leaving the laboratory, which would be consistent with the more widely reported positive relationship between perceived stigma and disordered eating patterns. To our knowledge, no studies have been conducted that explore rebound eating effects following laboratory-based studies in which participants restrict their intake, and while such research would provide logistical challenges, a better understanding of eating behaviour subsequent to participation in laboratory studies would be a useful addition to both the theoretical literature and perhaps also to the design of future eating behaviour research. Studies exploring the impact of perceived and internalised weight stigma on eating behaviour in a naturalistic setting could provide a more accurate picture of the relationship. A number of studies have used more ecologically valid techniques, such as ecological momentary assessment or daily diaries, to explore the relationship between experiences of weight stigma and self-stigmatising cognitions and eating-related outcomes in higher-weight individuals. For example, experienced and internalised weight stigma have been shown to negatively correlate with subsequent self-reported diet “healthiness” (Seacat et al., 2016) and reduced motivation to diet or lose weight (Vartanian et al., 2018). However, to date, there have been no studies reporting ecological assessment of the impact of weight stigma on actual eating behaviour. As cognitions and intentions do not necessarily translate into behaviour (Webb and Sheeran, 2006), future studies conducted in naturalistic settings should assess actual eating behaviours.

In contrast to high-weight individuals high in internalised weight stigma, those low in internalised weight stigma tended to eat more in the weight-stigma condition. The WBIS, and consequently its modified weight-neutral version, includes items capturing a complex mixture of cognitions and affect, many related to how people with higher-weight bodies interact with others or society as a whole (Meadows and Higgs, 2019). It is possible that higher-weight individuals with lower internalised weight stigma, who may not usually dwell on such issues, may react in unhelpful ways when reminded that society considers their bodies to be problematic. Alternatively, recent work on higher-weight individuals who reject and actively resist societal weight stigma have identified a negative relationship between weight stigma resistance and both weight-related self-devaluation and weight-related distress, including concerns about how others perceive one (Meadows and Higgs, 2018). Thus, the increased snack intake in high-BMI participants who were nevertheless low in internalised weight stigma may reflect psychological reactance and engagement in a form of behavioural resistance to the stigmatising material.

Among normative-weight participants, there was a tendency to eat less in the weight-stigma condition compared with the control condition, irrespective of levels of internalised weight stigma. This effect is consistent with previous findings by Major et al. (2014), and may also be a form of impression management – to clearly distinguish themselves from the stigmatised fat others depicted in the weight-relevant stigma prime. Another possibility is that it represents participants’ fear of fat. On being made aware of the negative interpersonal consequences experienced by higher-weight individuals, slimmer participants may be motivated to ensure that this fate does not befall them and so restrict their snack intake. A recent cross-sectional study among a weight-diverse sample (BMI *M* = 26.5 kg/m^2^, *SD* = 6.3 kg/m^2^) of 193 college students found that perceived weight stigma positively predicted maladaptive eating, in particular, dietary restraint, and that this effect was mediated by fear of fat (Wellman et al., 2018). While some population-based studies have demonstrated that stigmatising images in weight-related health campaigns have little effect on higher-weight individuals but do tend to increase healthy behaviour intentions in lower-weight individuals (Young et al., 2016), other studies have reported null effects on health behaviour motivation or implementation across the weight spectrum (Puhl et al., 2013; Simpson et al., 2017). Thus, from a practical viewpoint, stigmatising messages appear to have little to recommend them in terms of health promotion.

Importantly, despite internalised weight stigma often being considered to have similar effects in higher- and normative-weight individuals, perhaps differing only in degree, the present study indicated differential moderating effects of internalised weight stigma in response to stigma exposure dependent on participant weight status. Weight-related stigmatising experiences in Western society do not occur in a vacuum, but rather within a pervasively hostile anti-fat environment in which higher-weight individuals occupy a recognised subordinate status, complicated by aspects of blame and shame, with consequent implications for the inter- and intrapersonal dynamics of such interactions (Fiske, 2010; Barlösius and Philipps, 2015). Attributing negative treatment to prejudice is likely to be more onerous when it targets a stable, genuinely disadvantaged identity (Schmitt and Branscombe, 2002); the lived experience of a fat joke addressed at a very fat young girl, for example, may well not be equivalent to one addressed to a slim girl with body image issues. Studies that have independently assessed the effects of weight-related teasing across different weight groups have produced conflicting results: while all studies consistently report significantly higher frequency of weight-related teasing in heavier participants, some (e.g., Goldfield et al., 2010; Puhl and Luedicke, 2012) have found no difference in affective responses to victimisation by weight status, whereas others (e.g., Quick et al., 2013) found that heavier individuals reported greater distress as a result of weight-based victimisation than did slimmer individuals. Therefore, it should not be assumed that measures of stigma, whether experienced or internalised, are capturing the same qualitative experiences in higher-weight and normative-weight participants (Meadows et al., 2017).

The present study has a number of limitations. First, unlike previous studies using the EAH paradigm, participants were not fed to satiety in the lab but were asked to attend full. It is possible that participants were not sufficiently sated to obtain a true measure of EAH and that hunger may have been driving eating behaviour. However, baseline hunger levels confirmed the fed state in the majority of participants, and all analyses controlled for baseline hunger levels. Secondly, by using an interpersonal-relationship paradigm for the experimental manipulation, we aimed to eliminate the potential confounding by non-identity-related stress that may have been present in the study by Major et al. (2014), in which the vignettes discussed both employment and health problems associated with being higher-weight. However, while all participants in the present study were required to be non-smokers, thus ensuring the neutral control condition was non-personally relevant, it is not possible to rule out that some effects may have been driven by participants’ own health concerns becoming salient on reading about smoking, a behaviour known to be highly relevant to health. Such an effect may have translated into control participants eating fewer snacks, and reduced the size of any differences due to experimental condition.

Imbalances also occurred in the combination of high and low weight status and high and low levels of internalised weight stigma, which may have led to increased uncertainty around the estimates of effect size and reduced statistical power. From a methodological point of view, it is more difficult to recruit participants with high BMI and low internalised weight stigma, and low BMI but high internalised weight stigma than the reverse combinations, simply due to the relative prevalence of each in the general population. Future studies are needed to replicate this finding, and to test the hypothesised mechanisms driving differential responses among participants of different weight statuses and levels of internalised weight stigma. Online studies provide opportunities to strategically target individuals likely to endorse a broader array of weight-related attitudes, whereas a laboratory-based study is limited by geographical constraints. However, more complex experimental design would be required to achieve the effect of being observed in an online context. EMA studies with more targeted recruitment designed to capture this less common combination of high weight and low internalised weight stigma, for example by recruiting from the size acceptance community as well as from the general population, may be one solution to this problem. Further, the relative paucity of male participants made it impossible to test for gender differences in stigma response, and this should also be addressed in future studies.

Finally, internalised weight stigma was assessed as a trait-level variable prior to the lab-based phase of the study. While it is reasonable to expect that existing levels of internalised weight stigma will moderate how an individual responds to a stigmatising prime, it would be of interest to test the effect of the prime on state levels of internalised weight stigma, as well as the mediational effect of the prime on EAH via state internalised weight stigma.

## Conclusion

In conclusion, this is the first study to explore the role of internalised weight stigma on snack intake in response to a weight-relevant stigma prime. While the findings suggest a tendency for higher versus lower levels of internalised weight stigma to be associated with reduced energy intake in higher-weight individuals in the weight-stigma condition compared with a control condition, it is likely that at least part of this effect was a result of self-presentational motivation, and the possibility of a subsequent rebound effect on eating behaviour cannot be ruled out. Thus, it would be premature to suggest that experienced or internalised weight stigma may reduce intake in a natural environment. Although it could be argued that these findings support a potential role for the use of stigmatising content in health promotion messages, with the goal of encouraging reduced consumption, a growing body of research fails to support such an approach. Stigmatising public health messages have consistently been shown to have paradoxical effects, including increased desire for high-calorie foods (Tomiyama and Mann, 2013) and reduced self-efficacy for healthy behaviour change (Puhl et al., 2013; Simpson et al., 2017). Stigmatising public health messages have also been criticised on ethical grounds, for increasing anti-fat bias in society, for shifting focus away from the far more significant social determinants of health, and even for being inconsistent with a human-rights approach to health (O’Hara and Gregg, 2012; Pausé, 2017; Couch et al., 2018; Medvedyuk et al., 2018). Given the somewhat unexpected nature of the results, at least in terms of the extant literature on the relationship between internalised weight stigma and self-reported eating behaviour, further research is needed to replicate these findings and to elucidate the mechanisms underlying the processes involved.

## Ethics Statement

All subjects in this study gave written informed consent in accordance with the Declaration of Helsinki. The protocol was approved by the University of Birmingham Ethical Review Committee.

## Author Contributions

AM conceived the study and was responsible for data acquisition and analysis, and drafted the initial version of the manuscript. AM and SH contributed to the design of the study and interpretation of the data. Both authors were involved in critical revision of the manuscript, approved the final manuscript, and agreed to be accountable for all aspects of the work.

## Conflict of Interest Statement

The authors declare that the research was conducted in the absence of any commercial or financial relationships that could be construed as a potential conflict of interest.

## Footnotes

1 The term “higher-weight” is preferred over “overweight” or “obese,” as these latter medicalise body weight, and mark heavier bodies as “diseased,” even in the absence of any other biological perturbations (Meadows and Daníelsdóttir, 2016). Where specific BMI categories apply to participants in previously published studies, these terms have been reproduced in this manuscript within inverted commas. As the present study assesses the impact of a weight stigma versus control intervention between groups based on the distinct classification between perceived acceptability of their weight status (i.e., within or outside currently acceptable societal boundaries, and whether or not that weight is likely to be stigmatised), the term “normative-weight” (Tylka et al., 2014) is used to contrast with “higher-weight” to distinguish between the two groups.

2 http://Qualtrics.com

3 Other EAH studies have used intervals of 10–30 min.

4 Cheese crackers (Jacob’s Mini Cheddars, 80 g, 516 kcals and 29.5 g fat/100 g), crisps (salt and vinegar Pringles, 80 g, 512 kcals, and 32 g fat/100 g), pretzels (Penn State sour cream and chive pretzels, 80 g, 443 kcals and 12.9 g fat/100 g), chocolate (Mars M and Ms, 380 g, 485 kcals and 20.4 g fat/100 g), biscuits (ASDA Chosen by You milk chocolate oatie crumbles, 180 g, 497 kcals and 22.7 g fat/100 g, and sweet popcorn (Butterkist toffee popcorn, 80 g, 414 kcals and 8.4 g fat/100 g).

5 Of the 120 participants who stated their profession as “Student,” 86 (71.7%) were recruited through the School of Psychology; the remainder accessed the study via community recruitment advertisements.

6 Additionally, internal reliability was low for the EWS-3 (Kuder-Richardson’s α = 0.67). Despite the very low number of participants completing the SSI, Cronbach’s α was 0.97. Given the low number of participants who had completed this measure, data from non-completers were revisited. Including data from study non-completers, a total of 22 participants had completed the SSI, and of these, 95% endorsed at least one prior experience of weight stigma.

7 It was not possible to test if this effect differed by weight status due to low numbers of low-BMI male participants (*n* = 2 in the control condition, and *n* = 0 in the weight-stigma condition).

8 The unadjusted model is included in the Supplementary Materials. Only minor differences in regression coefficients and conditional effects were noted.

9 Running the analyses with self-classified weight as a continuous variable did not change the pattern of the results.
