# Supplementary material for: Internalised Weight Stigma Moderates the Impact of a Stigmatising Prime on Eating in the Absence of Hunger in Higher- but Not Lower-Weight Individuals
Source: Front Psychol. 2019 May 8;10:1022. doi: 10.3389/fpsyg.2019.01022 (PMC6519002; doi:10.3389/fpsyg.2019.01022)
Supplement: Supplementary file 1 [file Data_Sheet_1.PDF]

Supplementary Material

Internalised weight stigma moderates the impact of a stigmatising prime on eating in the absence of hunger in higher- but not lower-weight individuals

Angela Meadows,* **Suzanne Higgs**

Corresponding Author: drameadows@gmail.com

Vignettes used in experimental manipulation

It is well known that [obese people/smokers] are at increased risk of a wide range of health problems. But new scientific evidence suggests that there are costs in interpersonal relationships also.

Being [fat/a smoker] may have an especially negative impact on dating prospects. A recent experimental study asked 238 university students to rate a personal advertisement of a [man/woman] seeking a dating partner. They found that identifying the individual as [obese/a smoker] resulted in worse evaluations of the prospective dating partner by both women and men compared to an ad where [weight/smoking] wasn't mentioned. Also, including the information caused readers to assign more negative stereotypes to the individual, for example believing they were weak-willed, lazy, unintelligent, or lacking personal responsibility.

Other studies have found similar results. When 449 university students were asked to rank six pictures of hypothetical sexual partners, including [an obese partner / a smoker] and partners with various disabilities (including a partner in a wheelchair, missing an arm, with a mental illness, or described as having a history of sexually transmitted diseases), both men and women ranked the [obese person/smoker] as the least desirable sexual partner compared to the others. However, [men/women] ranked the [obese partner/smoker] as significantly less preferable than [women/men] did, suggesting that stigma may be heightened for [women/men] in sexual relationships. These findings parallel other work demonstrating that [obese] [women/men] [who smoke] are rated as being less sexually attractive, skilled, warm, and responsive, than [normal-weight/non-smoking] peers.

Martha Livingston of relationship counseling charity Relate says that more and more couples where one of the two is [fat/a smoker] are seeking help with their relationships. "The [weight/smoking] is often a cause of contention in the marriage," she says. "Either they say something and are thought of nagging, or they don't say anything and it just festers. But either way, relationships can suffer."

Table S1. Effects of experimental condition, internalised weight stigma, and weight status on eating in the absence of hunger – adjusted and unadjusted models

Only very minor differences in variable regression coefficients and conditional effects were noted in the unadjusted model compared with the full model (controlling for gender, experienced weight stigma, and baseline hunger). The model containing only experimental condition, internalised weight stigma, and weight status explained 6.9% of the variance in total energy intake. In the full model, gender explained a further 2.5% of the variance, experienced weight stigma 0.5%, and baseline hunger 18.5%. The variance explained by the three-way interaction was reduced by just 0.1% in the unadjusted model (full model change R^2 = .026, p = .028; adjusted model change R^2 = .025, p = .053).

The conditional effect of IWS in the stigma condition compared with the control condition remained significant in the high-BMI group: full model effect = -85, F(1,139) = 7.46, p = .007; unadjusted model effect = -88, F(1,150) = 7.79, p = .006. The conditional effect in the low-BMI group was non-significant in both the full and unadjusted models.

	B	*SE*	*t*	95% LLCI	95% ULCI	*p*
Constant	172	28	6.18	117	227	< .000
Vignette	-54	39	-1.36	-132	24	.176
IWS	6	21	0.29	-35	47	.774
Weight status	-11	40	-0.27	-90	68	.791
Vignette * IWS	-2	29	-0.07	-60	56	.943
Vignette * Weight status	81	60	1.36	-37	200	.177
IWS * Weight Status	19	27	0.71	-34	73	.482
Vignette * IWS * Weight status	-85	43	-2.00	-171	-1	.047

Unadjusted model, N = 158. Total R^2 = .069, F(7,150) = 1.56, p = .143. ΔR^2 with three-way interaction = .025, F(1,150) = 3.99, p = .047.

Unstandardised regression coefficients shown. Vignette coded 0 = Smoking, 1 = Weight; Weight status coded 0 = BMI < 25 kg/m^2, 1 = BMI ≥ 25 kg/m^2;
IWS = Internalised weight stigma.

Table S2. Bivariate correlations between internalised weight stigma and self-report eating behaviours in high-BMI and low-BMI participants

	BMI < 25 kg/m^2	BMI ≥ 25 kg/m^2
Current dieting	-.581***	-.110
DEBQ-Restraint	.623***	.153
DEBQ-Emotional eating	.237*	.407***
DEBQ-External eating	-.003	-.099
Eating Disorders Diagnostic Scale	.101	.164

Current dieting coded: 1 = Dieting for weight loss, 2 = Watching what I eat to maintain weight, 3 = Not dieting.
* $p < .05$, *** $p < .001$
DEBQ, Dutch Eating Behaviour Questionnaire
